# Professor Helmut Trimmel – Adapted Interview from the 3^rd^ Neurotrauma Treatment Simulation Center in Vienna, Austria

**DOI:** 10.25122/jml-2024-1012

**Published:** 2024-07

**Authors:** Stefana-Andrada Dobran, Alexandra Gherman

**Affiliations:** 1 RoNeuro Institute for Neurological Research and Diagnostic, Cluj-Napoca, Romania; 2 Sociology Department, Babes-Bolyai University, Cluj-Napoca, Romania

**Interviewee: Professor Helmut Trimmel**

**Interviewer: Stefana-Andrada Dobran**

Professor Helmut Trimmel specializes in anesthesiology and intensive care medicine and has served as the Head of the Department of Anaesthesiology, Emergency and Intensive Medicine at Wiener Neustadt Regional Hospital in Austria since 2000. He is also a Full Professor in Anesthesiology, Emergency, and Critical Care Medicine at the Danube Private University.

Professor Trimmel holds the position of Vice President of the Austrian Society for Anesthesiology and Intensive Medicine (ÖGARI) and is the Head of the Karl Landsteiner Institute for Emergency Medicine, Medical Simulation and Patient Safety. Furthermore, he serves as Deputy Medical Director of Christophorus Air Rescue.

His research interests include prehospital emergency medicine, traumatic brain injury, medical simulation, and patient safety. Prof. Trimmel is a member of numerous scientific societies and organizations, as well as the State Medical Council in Lower Austria.


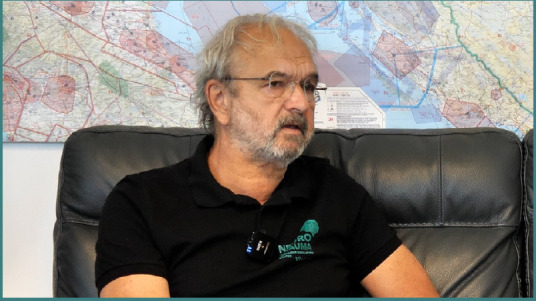


**S.D.: Dear Professor Helmut Trimmel, we are here at the third edition of the Neurotrauma Treatment Simulation Center (NTSC), a multidisciplinary hands-on program that aims to address global organizational issues to ensure optimal neurotrauma treatment. We have prepared a set of interview questions for you to offer a better understanding of multidisciplinary neurotrauma management to our audience and the way in which your expertise and involvement with the Academy for Multidisciplinary Neurotraumatology (AMN) has shaped your view on the matter. Firstly, what motivated you to be part of the NTSC experience and an active part of the AMN, and how you consider that your background in anesthesiology and intensive care medicine influences your perspective on neurotrauma management?**

H.T.: Thank you for this question! Neurotrauma affects mostly young people. It’s the number one killer for patients below 45 years of age, so I think this is a very important point that we face. This is a very big socioeconomic as well as individual burden. This motivated me because I think, especially for emergency medicine and intensive care, patients with severe traumatic brain injury are a group that needs high-quality care. This high quality of care needs many disciplines to give patients what they need, and this motivated me to be part of this neurotrauma course, which includes several medical disciplines.

**S.D.: What do you consider the main challenges in the current approach to neurotrauma and how do you think that the AMN can mediate these challenges?**

H.T.: I think neurotrauma is best treated if it starts in the early beginning. One of the major challenges is to give qualified care in the pre-hospital scene, especially by ambulance teams staffed with physicians who make drug-facilitated intubations, stabilize the hemodynamics, and bring the patient to a hospital that can provide neurotraumatological care in the OR (Operating Room), and the intensive care unit, especially if the transportation is by helicopter. This would be a high level of care. In this course, we discuss with colleagues from all over the world and we see a differentiated point of view and learn from each other – this makes this course so valuable.

**S.D.: How do you think that the AMN as an organization can help improve the management worldwide?**

H.T.: As I said, it brings together people from different systems of care from all over the world, and you can learn from each other – they can see what we do and we see what they do. There are very highly sophisticated systems like South Korea, for example, and the European Union, and there are also participants from countries that do not have all these possibilities. I think this makes it very valuable — to come and discuss this together.

**S.D.: Could you describe a case with a challenging decision you had to make regarding the treatment of a neurotrauma patient?**

H.T.: Yes, I remember a young guy who had a fall from height. He was a mountain climber, brought by helicopter after a very difficult rescue operation. It was a very stormy winter day and they needed a lot of time to get him out of there. He was brought to a smaller hospital and they first performed stabilizing spinal surgery, then called the university hospital, which refused to take over the patient due to futility. They called us and I talked to them and said “In this case, we will take care of this patient because we are the hospital responsible for his place of residence.” When he arrived he was in a really bad condition and we had a discussion with the neurosurgeon, with the radiologist, with the trauma surgeon, and with us as intensivists. We came to the point that we said “We give this patient a chance, one last try to save his life.” Now, he’s walking by himself. He’s not able to work, but he’s living at home with his parents, with his family, and he’s happy to live. I see him once a year for close-up visits and I’m very happy that we took this chance. I will always remember this guy because we really saved his life and he has a very good quality of life in respect to the lesions that he had.

**S.D.: As you mentioned multidisciplinary teamwork during this case, what are the key factors that contribute to a successful collaboration in the treatment of neurotrauma?**

H.T.: The point is, you have to have a rescue chain — as I may address it — from the beginning, in the field, to the intensive care unit, to the OR, to the rehabilitation center. All these guys have to talk to one another and make a clear treatment plan, to give the patients the best chance to survive; not only to survive but to come to a quality of life that is worth living. I think it’s not so necessary to talk about mortality and survival, but it’s more important to talk about the quality of life and to give all the measures that enhance this quality of life because, in the end, this is what you, as a patient, will look at.


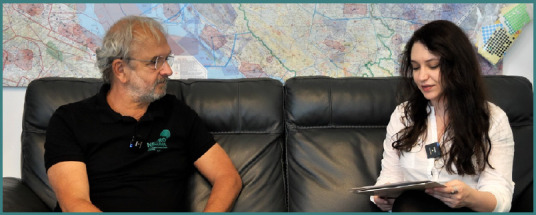


**S.D.: What are the key takeaways or skills the participants from NTSC should gain, and what are your future expectations regarding multidisciplinary neurotrauma management?**

H.T.: I think the key takeaways from this course are the [different] points of view that people from all over the world have regarding neurotrauma management. The main point is to learn that close working together will give the patient the best chance. If you take this with you and some examples of how to organize this, then you have a lot to bring home and set up something similar at your hospital. On a routine basis, to sit together to talk through the patients, to make the treatment plan from the beginning, in the ICU, to the rehabilitation (first early rehabilitation in the ICU) – this makes the difference in the quality of life in the end.

**S.D.: Do you have a final message on your behalf as NTSC faculty?**

H.T.: I’m happy to be part of this faculty. I welcome colleagues from all over the world who are coming to us. I think, based on the feedback from the last two courses, that there are many things that the colleagues take with them. I think we should do this further on and expand it on a broader basis in the future.

**S.D.: Thank you very much!**

H.T.: Thank you for the interview!

